# An unexpected case of tetanus in a fully immunized 20-year-old female: a case report

**DOI:** 10.1186/s12245-024-00633-1

**Published:** 2024-04-24

**Authors:** Mitsutoshi Okuda, Atsushi Morizane, Sunao Asaba, Saika Tsurui, Ryutaro Tsuno, Mariko Hatakenaka, Tomoko Sugimura, Yuichi Saisaka

**Affiliations:** Critical Care and Emergency Center, Kochi Health Sciences Center, Ike 2125-1, Kochi City, Kochi 781-8555 Japan

**Keywords:** Tetanus, Vaccination, Vaccinated

## Abstract

**Background:**

Widespread vaccinations have significantly decreased the number of tetanus cases in developed countries. Today, most cases of tetanus affect the elderly and those with inadequate immunization in developed countries such as Japan. As vaccinations were believed to be nearly 100% effective in preventing tetanus, tetanus in young, immunized individuals were considered unlikely. However, unexpected tetanus infection has been reported in young adequately immunized individuals.

**Case:**

We herein describe a 20-year-old immunized female who visited our emergency department with trismus and painful muscle spasms that started after receiving a puncture wound to her right foot. A physical examination revealed an elevated body temperature (38°C), trismus, muscle spasms in her right leg and neck, and a puncture wound at the sole of her right foot. Following the development of dyspnea after admission to the intensive care unit, the patient was intubated and mechanically ventilated. She fully recovered after six days in intensive care.

**Conclusion:**

The present case serves as a stark reminder that tetanus may still occur in young, immunized individuals. Patients with a history of immunization may have a better prognosis than those with no immunizations.

## Background

Although widespread vaccinations have significantly decreased the number of tetanus cases in developed countries, it still affects the elderly and those with incomplete immunization [1, 2]. In Japan, the majority of new tetanus cases occur in the elderly with inadequate vaccination [3]. Tetanus in young patients is extremely rare since tetanus toxoid vaccination became mandatory in 1968. However, unexpected tetanus infection has been reported in young, immunized patients. In some cases, patients’ levels of tetanus antibodies were higher than the protective value. We herein report a case of tetanus in a 20-year-old female who received all necessary vaccinations during childhood and made a full recovery after intensive treatment.

## Case presentation

**Fig. 1:**
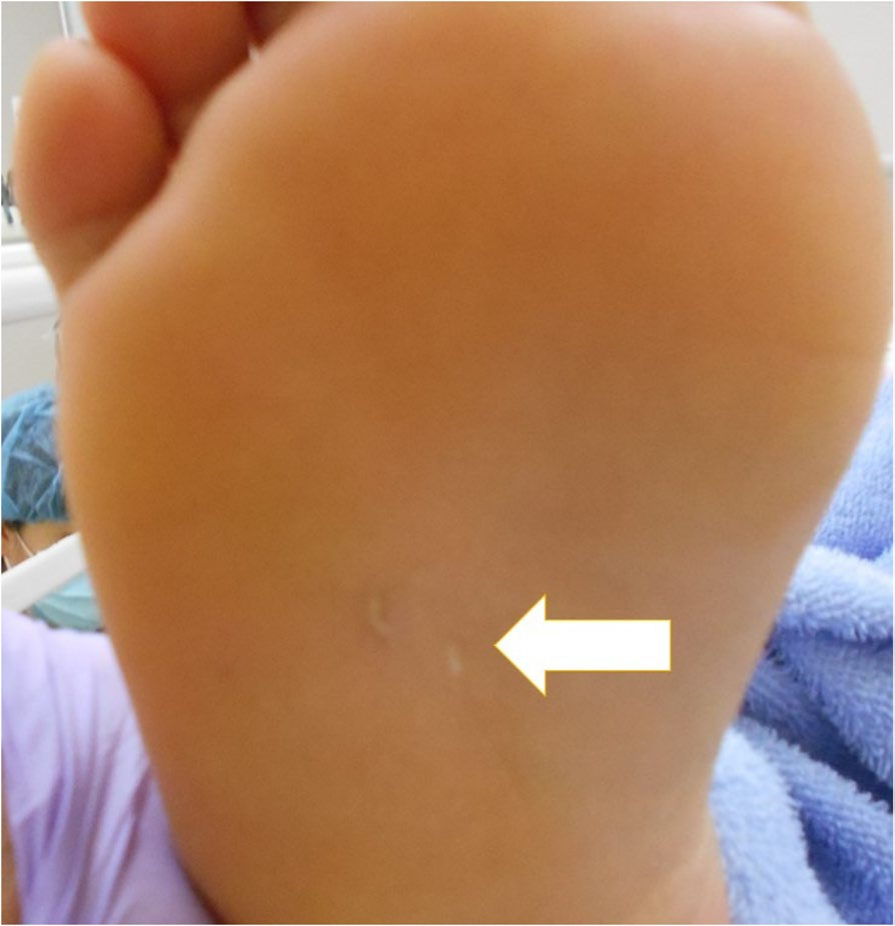
Puncture wound in the right foot. Legends: The patient stepped on a rusty nail in the fields 5 days prior to her visit. Physical examination revealed a puncture wound at the sole of her right foot

The patient was a 20-year-old female who visited our emergency department after developing fatigue, difficulty opening her jaw, and stiffness in the back of her neck. A physical examination revealed an elevated body temperature (38°C), trismus, muscle spasms in her right leg and neck, and a puncture wound at the sole of her right foot (Fig. 1). The patient reported stepping on a rusty nail while in the fields 5 days prior to her visit.

Her medical records showed that she had been fully vaccinated with the diphtheria-tetanus-pertussis (DTaP) vaccine at the age of 6^th^, 7^th^, and 8^th^ month and received a booster DTaP vaccine at the age of 22^nd^ month. This vaccination was in accordance with the Japanese Immunization Act.

A further examination showed no evidence for other conditions that may cause similar symptoms, including cerebrospinal infection, mandibular disorders, oral disorders, and pharyngeal disorders. The patient’s history and symptoms were highly suggestive of tetanus. Tetanus immunoglobulins and toxoid were administered at the emergency department after tetanus was diagnosed.

Following the development of dyspnea after admission to the intensive care unit, the patient was intubated and mechanically ventilated. She was orally intubated after deep sedation and muscle paralysis. The patient was successfully extubated after 6 days in intensive care. She was able to open her mouth with ease and no muscle spasms were present. She was discharged 11 days after admission with no further complications.

## Discussion

We encountered a case of unexpected tetanus infection in a 20-year-old fully immunized female who made a quick and complete recovery after 6 days in intensive care. This case highlights two important points. Tetanus may still affect the young adequately immunized individuals, and symptoms may be milder and recovery may be more rapid in individuals with previous immunization than in those with no history of immunization.

The present case demonstrates that even with an adequate vaccination status, young individuals may be affected by tetanus. Most tetanus cases in Japan occur in the elderly. In Japan, 499 cases of tetanus were recorded between July 2010 and March 2016. The median age of these patients was 74 years old, and only 58 patients were younger than 50 years old [4]. The elderly population in Japan is at a higher risk of contracting tetanus because tetanus toxoid vaccines were not mandatory until 1968 [3]. A national epidemiological study in 2018 revealed that less than 35% of individuals older than 50 years old had a toxin antibody level higher than 0.1 IU/ml, a value considered to be protective against tetanus infection. In contrast, 83% of those aged between 0–49 years had a toxin antibody level higher than 0.1 IU/ml [5]. Since tetanus vaccination became mandatory in 1968, the majority of the young population has been immunized, and as toxoid immunizations were considered to be almost 100% effective in preventing tetanus infection [6], tetanus in a young immunized patient was considered highly unlikely and rare.

We found several case reports of tetanus in immunized young patients [6–8]. This type of case is estimated to occur in approximately 4 out of 100 million vaccinated individuals with adequate immunity [9]. Furthermore, tetanus infection has been reported in patients with adequate amounts of antitoxin antibodies [10–12]. Vollman et al. [10] described a case of tetanus in a 31-year-old male with an antitoxin antibody level higher than the protective value (0.1 IU/ml). A systematic review of tetanus despite previous immunization by Hopkins et al. [13] revealed 359 cases of tetanus in patients with either a prior receipt of at least one toxoid vaccination or an antibody titer above the protective value [13].

The exact reason why tetanus occurs in immunized individuals is unknown. Suggested theories include the toxin overwhelming host defenses, antigenicity variations between the toxin and toxoid, and a suppressed immune response [9]. Another theory is that tetanus toxoid vaccination may not generate immunity in some subjects. Crone et al. [14] reported three cases of grade 3 tetanus that occurred in immunized patients. One of the patients had adequate titers of toxin antibodies (0.2 IU/ml); however, an in vivo mouse protection bioassay revealed a titer lower than 0.01 IU/ml. The authors theorized that while the patient had immunity against tetanus toxoid, there may have been a lack of immunity against tetanus neurotoxin [14].

Currently, the WHO recommends a primary series of 3 doses of tetanus toxoid containing vaccine with the first dose administered from 6 weeks of age and the third dose be completed by 6 months of age if possible. The WHO also recommends providing 3 tetanus toxoid containing vaccine booster doses at 12–23 months of age, 4–7 years of age, and 9–15 years of age with 4 years of interval between the booster doses [15].

Tetanus vaccination provide adequate long lasting immunity against tetanus in the majority of the recipients. Gao et al. [16] conducted a meta-analysis of 42 studies to investigate the durations of protective immunity against diphtheria, tetanus, pertussis, and polio. The authors included studies that examined long-term vaccine efficacy, geometric mean titers or geometric mean concentrations, and the percentage of seroprotection after the administration of vaccines to healthy subjects. The authors analyzed 16 studies and found that nearly all subjects had an antibody level higher than the protective value (> 0.1 IU/L) 10 years after vaccine administration. Hammarlund et al. [17] performed a cross sectional analysis of serum antibody titer in 546 adults who were aged between 19 to 87 years old to study the duration of immunity following vaccination against smallpox, tetanus, and diphtheria. In this study, the authors found that around 97% of the population was seropositive to tetanus with the mean titers of 3.6IU/ml. The authors defined the half time of tetanus specific antibody as a function of time after last vaccination and found that the overall half time to be 14 years. The authors also created a mathematical model which combined antibody magnitude and duration and predicted that 95% of the study population will remain seroprotected against tetanus for up to 72 years without further booster vaccination [17]. However, the metanalysis of 42 studies by Gao et al. [16] also found that approximately 7% of subjects did not had a protective antibody level 2–3 years after vaccination. It can be said that although tetanus vaccination provides long-term immunity to the majority of individuals, it appears to fail in some.

For those with unexpected failure of immunity against tetanus after primary vaccination, booster doses in the adulthood may provide adequate immunity. A single booster dose of 1.9Lf of tetanus toxoid was effective in providing adequate immune response according to 2 studies that involved health care workers and elderly in Sweden [18, 19]. A study that investigated the efficacy of tetanus booster immunization in 680 army recruits in China showed that a single dose of booster was calculated to maintain protection for around 22 years [20].

However, the efficacy of implementing adult booster vaccination programs is debatable. In the United State, adult booster vaccination scheduled was recommended once every 10 years since 1964 [21, 22]. Countries such as Germany, Finland, and Canada also recommend booster vaccination every 10 years [22]. On the other hand, WHO currently does not recommend adult tetanus or diphtheria booster vaccinations for individuals who have completed childhood vaccinations. This is supported by studies that found no benefits of providing regular adult tetanus booster vaccinations. Slifka et al. [22] compared the incidence of tetanus between 2 groups of countries. One group was consisted of 21 countries that vaccinated adults with tetanus boosters every 5 to 20 years while the other group consisted of 9 countries that did not recommended booster vaccinations for those 20 or older. The authors found no significant decrease in tetanus incidence rates in countries that provided booster vaccinations to adults every 5 to 20 years [22].

We believe that implementation of regular adult booster vaccinations should be based on each countries historical backgrounds of vaccination programs. Countries with a certain number of population who did not recieve mandatory tetanus vaccinations such as Japan and Finland would benefit from recommending regular adult booster vaccinations [23]. Together with the fact that primary tetanus vaccination do not generate adequate immunity in some recipients, we believe the implementation of regular adult tetanus booster vaccinations do have benefits in the prevention of tetanus in certain countries like Japan.

Our patient was successfully extubated after being intubated and mechanically ventilated for 6 days and was discharged with no complications, demonstrating that tetanus patients with previous vaccinations may achieve a better prognosis than those with no immunization. The duration of mechanical ventilation in the present case was significantly shorter than the median duration of mechanical ventilation among tetanus patients in Japan between 2010 and 2016 (mean = 23 days) [4]. A systematic review of tetanus despite previous immunization by Hopkins et al. [13] suggested that those with prior immunization have a higher survival rate and better prognosis. The authors found that 180 out of the 210 cases with reported clinical outcomes survived to discharge. The majority of patients who were followed after discharge showed the complete resolution of their symptoms [13].

The principles of the management of tetanus are neutralization of tetanus toxins, prevention of production of tetanus toxin by using antibiotics and wound debridement, minimization of the effect of the toxin in the central nervous system [1, 7].

Neutralization of circulating tetanus toxin is achieved by administering immunoglobulins. The effect of tetanospamin that has already entered the central nervous systems cannot be reversed. Therefore, administration of immunoglobulins aims to neutralize circulating tetanus toxins before it enters the central nervous systems [1, 24]. Traditionally, 3–6000 units of immunoglobulin were administered intramuscularly [24]. Though in recent years, intrathecal administration of immunoglobulins have been shown to be more beneficial compared to intramuscular administration [25, 26]. Patients who received intrathecal administration of antitetanus immunoglobulin was reported to have shorter duration of spasms, shorter hospital stay, and shorter respiratory assistance compared to intramuscular administration [25]. A meta-analysis of published randomized studies including 12 clinical trials and 942 patients by Kabura et al. [27] compared intrathecal therapy and intramuscular therapy and reported that intrathecal therapy was superior in terms of mortality with no neurological side effects [27]. However the authors have also stated that the evidence is still insufficient for its use [27]. A rare complication of reversible paraplegia after high dose intrathecal administration of human gammaglobulins has been reported by Robert et al. [28]. High dose of immunoglobulin or drug preservative were attributed to this complication. Also, the authors mentions that as intrathecal administration of immunoglobulins are implemented in patients with severe tetanus with impaired consciousness, the complication can be hard to notice [27, 28]. Overall, intrathecal administration of antitetanus immunoglobulins can be a promising treatment for tetanus but still lacks definitive evidence. Intrathecal administration of immunoglobulins is not a standard practice in Japan but this may change in the coming years with more substantial evidence.

Controlling the source of infection is achieved through wound debridement and administration of antibiotics. Early debridement of infected wounds and removal of foreign bodies is crucial in preventing further toxin production [24]. The mainstay antibiotics of choice includes metronidazole or penicillin. Some favored metronidazole over penicillin because it was associated with convulsions as it produces a non-competitive voltage-dependent inhibition of GABA-A receptor [24, 29]. However, a randomized controlled trial by Kumar et al. studied the outcome of 161 tetanus patients after administering either a intramuscular benzathine penicillin, enteral metronidazole, or intravenous benzyl penicillin and found no significant difference in terms of length of hospital stay, incidence of dysautonomia and nosocomial pneumonia, and number of in-hospital deaths [30]. It can be said that penicillin is as effective and safe to use for tetanus as metronidazole. Erythromycin, tetracycline, chloramphenicol, and clindamycin are accepted as alternatives [1, 24, 29].

Sedation is the first-line treatment for controlling rigidity and spasms and also for autonomic dysfunctions [7, 29]. In terms of the control of rigidity and spasm, sedation with benzodiazepine such as diazepam or midazolam is widely used [1, 29]. Other choices for sedation are anticonvulsants such as phenobarbitone, phenothiazine such as chlorpromazine, and propofol. In cases in which sedation alone is inadequate to control rigidity and spasm, neuromuscular blocking agents and mechanical ventilation will be required [24, 29]. Long acting agent such as pancuronium and vecronium are widely used. Although evidences are lacking, the use of dantrolene and intrathecal baclofen were reported to be successful in treating rigidity and muscle spasms [1, 29]. The management of autonomic dysfunction can vary with no definitive evidence. Sedation is the first-line treatment with benzodiazepine, anticonvulsants, and morphine commonly used [24, 29]. Magnesium sulphate are used to reduce autonomic disturbances in artificially ventilated patients and to control spasms in non- ventilated patients [1, 7]. The use of magnesium sulfate was found to be effective in reducing spasms when used together with diazepam, lead to better control of dysatutonomia, reduced the need for mechanical ventilation, and lead to shorter hospital stay in a systematic review by Nepal et al. [31].

In this case, we swiftly administered immunoglobulins intramuscularly in the emergency department after making a preliminary diagnosis of tetanus after reviewing the patient history and physical examination findings. During the patients stay in the intensive care unit, we sedated the patient by administering fentanyl, dexmedetomidine, and midazolam. We also administered metronidazole to control infection and magnesium sulfate to control spasms and autonomic dysfunction. No apparent signs of severe spasms or autonomic dysfunctions were observed during her stay in the intensive care unit. We believe that swift administration of immunoglobulins and thorough measures to control infection, spasms and autonomic dysfunction together with the patient being fully immunized by completing primary tetanus vaccination during the first year of life contributed to her excellent recovery and prognosis.

The limitation of the present case is that we failed to measure the patient’s antitoxin antibody level. The measurement of serum tetanus antitoxin antibody levels is a rare practice in Japan. After encountering this case, our department established a new protocol for patients suspected of tetanus infection in which blood samples are sent to specialized institutions to measure tetanus antitoxin antibody levels.

## Conclusions

Tetanus may affect adequately immunized individuals and, thus, needs to be suspected in patients exhibiting trismus and painful muscle spasms, even with a complete vaccination history. Patients with a history of immunization may have a better prognosis than those with no immunizations.

## Data Availability

No datasets were generated or analysed during the current study.
